# Defining the Current Deployment of Neonatal Infusion Pumps in Low- and Lower-Middle-Income Countries: A Rapid Review

**DOI:** 10.1177/2333794X221127489

**Published:** 2023-01-09

**Authors:** Oliver Norton, Prashant Jha

**Affiliations:** 1 King’s College London, London, UK

**Keywords:** infusion pump, neonate, newborn, medical equipment, developing countries

## Abstract

There has been limited review of the reported deployment of infusion pumps in low- and lower-middle-income countries. This paper aims to identify the current distribution of infusion pumps in low- and lower-middle-income countries (LLMICs) used to treat neonates. A rapid review was conducted using material sourced from *ProQuest, Pubmed, Web of Science*, and *IEEE Xplore*. Twenty-six search results met the inclusion criteria. Within these, 41 neonatal healthcare facilities were discussed with 17 of the facilities having infusion pumps available, 13 limited access, and 11 none. Infusion pump use remains limited in Sub-Saharan Africa so efforts should be made to deploy specialist neonatal care improvement packages, potentially including infusion pumps designed for LLMICs. The effects of COVID-19, to neonatal care LLMICs, should be accessed to ensure progress has not regressed. These proposals aim to aid in the continued improvement of neonatal care globally and reduce newborn mortalities.

## Background

### Rationale

The global under provision of neonatal and maternal care is well documented. Despite several campaigns,^1-3^ UNICEF estimated that, in 2019, 30 million^2^ newborns were at risk, with 2.5 million neonates dying in the first 28 days of life.^2^ Neonatal mortalities are also the largest cause of disability-adjusted life years (DALYs) globally.^4^ As a result, the world is due to miss the third Sustainability Goal^2^ (SDG): “to ensure healthy lives and promote well-being for all at all ages.” At the current rate, the 81 countries with the highest newborn mortality rate will only achieve 18 neonatal mortalities per 1000 live births by 2030, rather than the goal of 12. The UNICEF “Survive and thrive: transforming care for every small and sick newborn” campaign^2^ has called this the “survival gap.”

The campaign identified several approaches which may close the “survival gap” including improved follow-up care, better infection control and, notably, higher quality of care. Analysis of the gap suggested that by implementing quality, specialist intensive neonatal care, newborn mortalities could be reduced by 28%^2^ by 2025. Stillbirths, maternal and neonatal deaths could also be decreased by another 19%.^2^

UNICEF and NEST360°, a global alliance aiming to reduce newborn mortalities in African hospitals, propose.^5,6^ that medical devices should be more widely deployed as part of providing higher quality care, such as for thermal management, respiratory support, and infection control and prevention. To improve infant hydration, nutrition and drug delivery, infusion pumps were identified.^5,6^ as a key medical device. The WHO also recognizes infusion pumps as a “priority medical device,”^7-9^ with the potential to improve the quality of patient care.^9,10^

The absence of medical devices generally in low- and lower-middle-income countries (LLMICs) has previously been discussed.^6,9,10^ However, there has been limited review literature discussing the current usage of infusion pumps specifically to treat neonates. This review focuses on the current distribution of infusion pumps in LLMICs used to treat neonatal patients. From this, the extent that they are being successfully deployed can be evaluated and help direct efforts for future investment into infusion pump usage.

### Infusion Pumps

The WHO defines infusion pumps as: “devices used to accurately deliver liquids through intravenous (IV) or epidural routes for therapeutic or diagnostic purposes.”^11^ The U.S. Food and Drug Administration categorizes infusion devices into different types^12,13^ including large volumetric pumps (LVP) and syringe pumps/drivers.

LVPs electromechanically deliver solutions, at a constant and controlled rate between 0.1 and 3600 mL/hour,^11,14^ from infusion bags. The mismanagement and inaccuracies of basic gravity-driven IV infusion set-ups can result in co-morbidities such as over- or dehydration^15^ of patients, leading to further complications. LVPs remove the need for frequent roller clamp adjustments^16^ in gravity-driven IV set-ups. LVPs are often referred to by the overarching term infusion pumps.

Syringe pumps deliver small volumes, ≤60 mL,^11^ of medication or fluids by pushing a syringe at a controlled rate to accurately deliver medications and fluids. Syringe pumps reduce the need for frequent small-dose injections of therapies. Syringe pumps are also referred to as syringe drivers.

Controlled infusions mean patients benefit from greater equity and safety; 2 of the WHO’s elements of healthcare quality.^7^ Additionally, by automating some processes, the pumps free up clinical staff’s time, improving treatment efficiency; a further element of the WHO’s health care quality measures.^7^ With 83 countries falling below the minimum WHO threshold of 22.8 skilled healthcare professionals per 10 000 population,^17^ efficient use of clinically trained staff is vital.^18^

## Methods

### Eligibility Criteria

To define the current use of LVPs and syringe pumps in LLMICs, a rapid review of recent literary material was conducted. The search aimed to isolate the use of LVPs and syringe pumps in LLMICs to treat neonatal or pediatric patients. Results using the terms “infusion pump,” “syringe pump” or “syringe driver” were included in the results.

Results also had to discuss countries that are LLMICs. LLMICs were determined using the World Bank database.^19^ The search terms “low-income countr*” and “middle-income countr*” were used to include these results. The search term “middle-income,” rather than “*lower-middle-income*,” was used to collect a greater range of search results. The asterisks were used to collect search results that referred to both country and countries. The term “*low-resource setting*” was also used so that the current use of infusion pumps in the most deprived healthcare settings could be included. Furthermore, results must also discuss “*neonatal*” or “*pediatric*” care. The search term “*pediatric*” was also used to ensure that both the UK and American spelling of the word were included in the search. The term “*newborn*” was also included to collect more results. Although not included as a search term, results that discussed obstetrics or maternal care were also included as this also affects prenatal and neonatal care.

It was important to isolate LVPs and syringe pumps in the search and exclude other types of pumps. Insulin pumps are another type of widely used fluid pump. Thus, “*NOT insulin*” was included as a search term to exclude them from the search results. Results relating to the use of infusion or syringe pumps for non-medical purposes were also excluded.

Collectively, this resulted in the search term: (infusion pump) OR (syringe pump) OR (syringe driver) AND ((low-resource setting) OR (low-income countr*) OR (middle-income countr*)) AND (neonatal) AND (newborn) AND ((pediatric) OR (pediatric)) NOT (insulin).

The search included academic papers and reports and excluded other types. Results not written in English, or adequately translated, were also excluded. To determine the most current use of infusion and syringe pumps, only material released in the last 5 years was included. The search window was decided to be 1/4/2017 to 1/4/2022.

Additional filters were used on 3 of the information sources (Table 1) to further improve the search. The filters were subject filters determined by the sources so are not the same for each search.

**Table 1.** Additional Search Filters.

Source	Additional filters
ProQuest	“patients,” “medical equipment,” “medical device industry,” “drug dosages,” “healthcare industry,” “hospitals,” “drugs,” “drug delivery systems”
Pubmed	-
Web of Science	“pediatrics,” “critical care medicine,” “health policy services,” “obstetrics gynecology,” “engineering multidisciplinary,” “emergency medicine”
IEEE Xplore	“biomedical equipment,” “pumps,” “patient treatment,” “health care,” “drug delivery systems,” “drugs,” “medical control systems,” “patient care”

### Information Sources

Four databases were used to conduct the review: *ProQuest, PubMed, Web of Science*, and *IEEE Xplore*.

### Search Strategy

The search strategy followed a repeated process for each database. Each criteria was applied in turn and then the number of results recorded in Microsoft Excel. Firstly, the search term was entered into the search engine and the number of results recorded. The date exclusion criteria was then applied, followed by the language exclusion criteria, the material type and finally the additional subject filters determined by the databases (Table 1).

### Selection Process

The selection process involved rapidly screening the title of each result and selecting those that the reviewer deemed potentially relevant to the search. Once all results from the database had been rapidly reviewed, all unselected results were discarded.

### Data Collection Process

Results were screened by an individual researcher. The data collection process involved the initially selected results being saved to the reference manager *Mendeley*. Each selected result was then read in full. Results which were initially selected but then deemed not to meet the inclusion criteria, upon reading, were then excluded.

Material was synthesized by first grouping the results by the global region. The global regions used were those defined by The World Bank.^20^ From this, the healthcare facilities, discussed in each result, were identified. Where the same facility was discussed in multiple papers, information was synthesized from the most recently published results. Finally, the availability and usage of pumps at each facility was identified. The results of this analysis were then compared so that the current distribution of infusion pumps in LLMICs could be determined.

## Results

**Figure 1.**
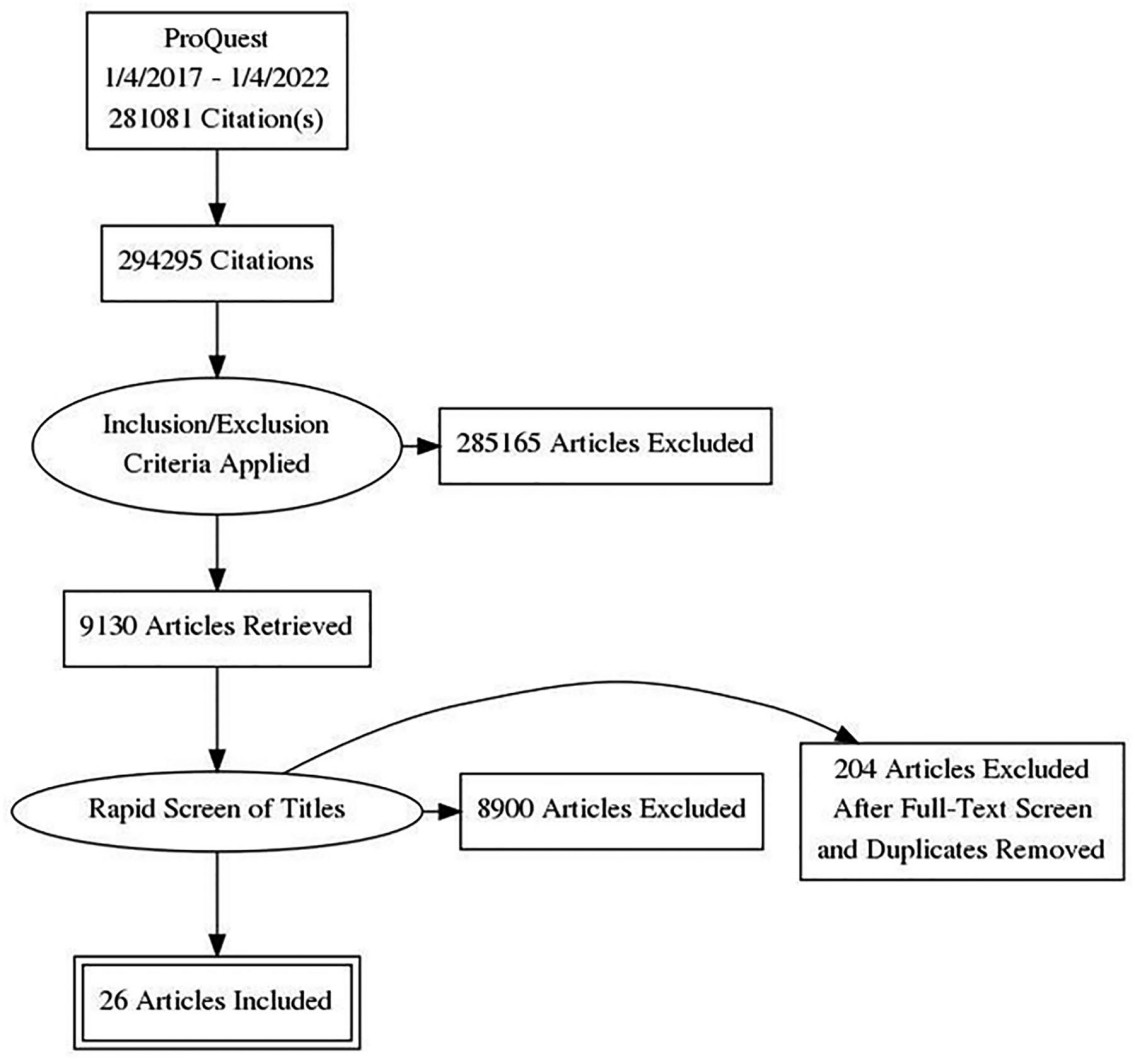
PRISMA flow diagram.

**Figure 2.**
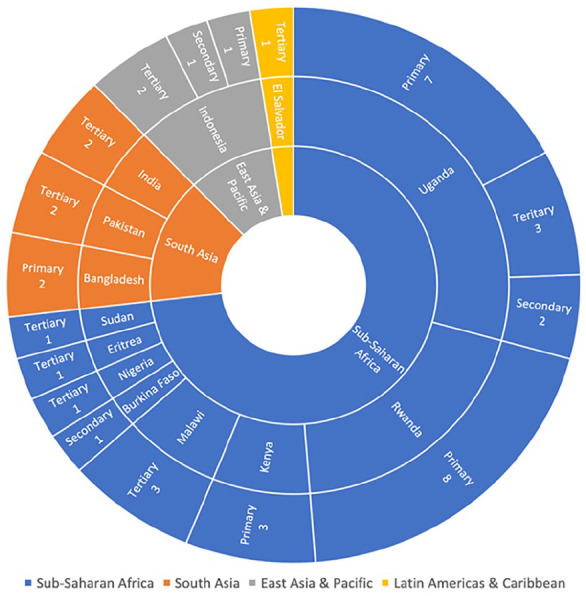
Global distribution of healthcare facilities identified in the search results.

**Figure 3.**
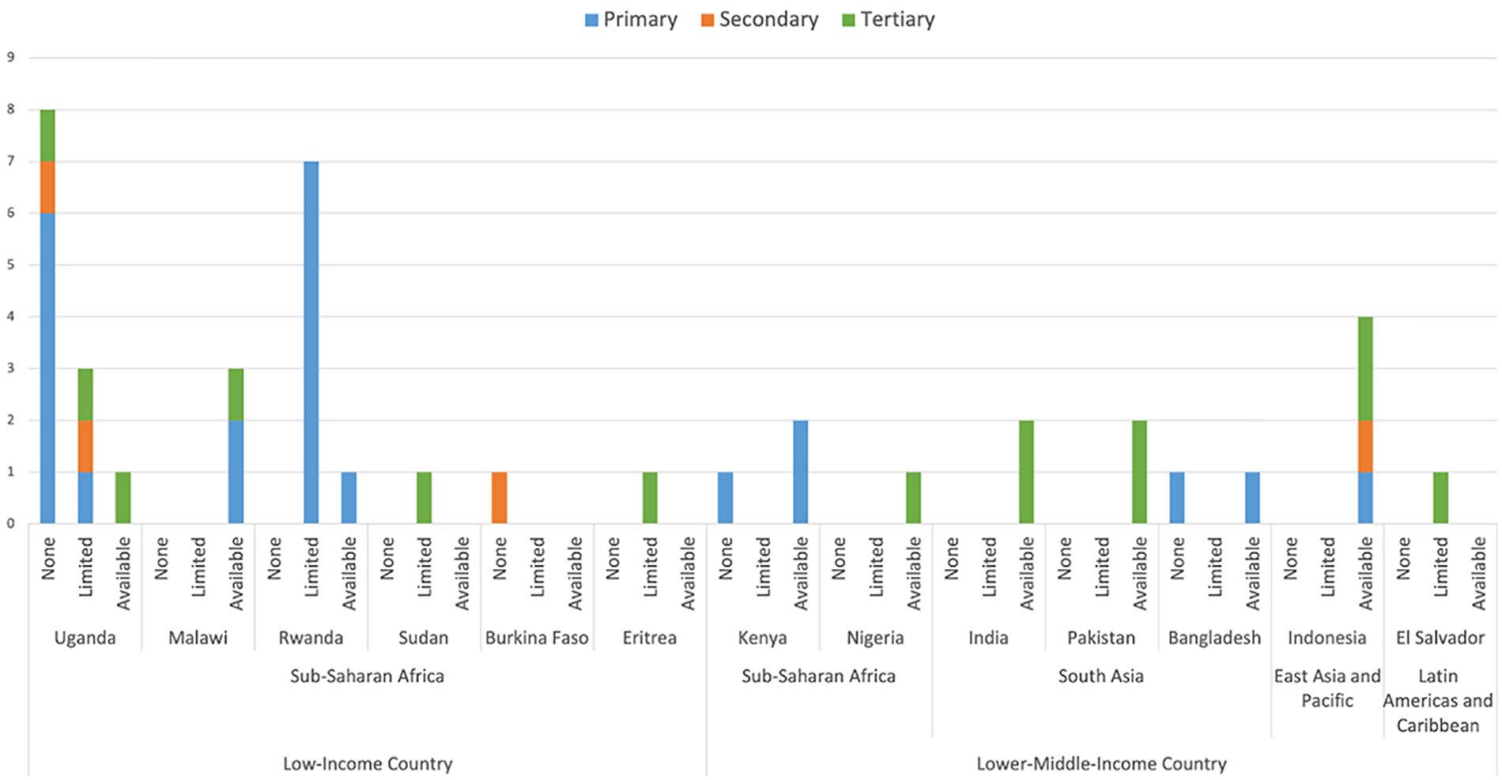
Infusion pump availability by location and facility level identified in the search results.

The search yielded 26 search results (Figure 1) which met the inclusion criteria (Table 2): 15 academic papers and 11 business reports. Five of the academic papers reported neonatal and maternal admission rates, causes and results. Another 5 discussed the design of novel infusion pumps. Four papers evaluated current care practices through equipment inventories, while 1 reported the results of a clinical trial. Seven of the papers discussed quality improvement strategies. Three of the results were multi-region studies, 8 discussed Sub-Saharan Africa countries, 6 South Asian, 1 East Asian and Pacific, and 11 Latin Americas and Caribbean. The 11 business reports were quarterly reports, published by Fitch Solutions Group Limited, detailing the current healthcare business landscape in El Salvador. Forty one healthcare facilities were identified in the search results. Thirty of the healthcare facilities were in Sub-Saharan Africa, 6 in South Asia, 4 in East Asia and Pacific, and 1 in the Latin Americas and Caribbean (Figure 2). In Sub-Saharan Africa, 10 of the healthcare facilities had no access to infusion pumps, 12 had limited access, and 8 had access (Figure 3). Whereas, in South Asia, 7 facilities had access to infusion pumps, while only 1 had no access to them. Similarly, all 4 facilities in East Asia and Pacific had access to infusion pumps. The 1 facility in the Latin Americas and Caribbean had limited access. Uganda was the most discussed country.

**Table 2.** Search Results.

Author(s)	Title	Publication	Publication year	Date last accessed	Source
Narayanan I, Nsungwa-Sabiti J, Lusyati S, Rohsiswatmo R, Thomas N, Kamalarathnam C, Wembabazi J, Kirabira V, Waiswa P, Data S, Kajjo D, Mubiri P, Ochola E, Shrestha P, Choi H, Ramasethu J	Facility readiness in low and middle-income countries to address care of high risk/ small and sick newborns	Maternal Health, Neonatology, and Perinatology	2019	08/04/2021	ProQuest
Fitch Solutions Group Limited	El Salvador Pharmaceuticals & Healthcare Report—Q1 2021	Fitch Solutions Group Limited	2021	08/04/2021	ProQuest
Fitch Solutions Group Limited	El Salvador Pharmaceuticals & Healthcare Report—Q2 2021	Fitch Solutions Group Limited	2021	08/04/2021	ProQuest
Fitch Solutions Group Limited	El Salvador Pharmaceuticals & Healthcare Report—Q1 2020	Fitch Solutions Group Limited	2020	08/04/2021	ProQuest
Fitch Solutions Group Limited	El Salvador Pharmaceuticals & Healthcare Report—Q2 2020	Fitch Solutions Group Limited	2020	08/04/2021	ProQuest
Fitch Solutions Group Limited	El Salvador Pharmaceuticals & Healthcare Report—Q3 2020	Fitch Solutions Group Limited	2020	08/04/2021	ProQuest
Fitch Solutions Group Limited	El Salvador Pharmaceuticals & Healthcare Report—Q3 2020	Fitch Solutions Group Limited	2020	08/04/2021	ProQuest
Fitch Solutions Group Limited	El Salvador Pharmaceuticals & Healthcare Report—Q4 2020	Fitch Solutions Group Limited	2020	08/04/2021	ProQuest
Fitch Solutions Group Limited	El Salvador Pharmaceuticals & Healthcare Report—Q1 2019	Fitch Solutions Group Limited	2019	08/04/2021	ProQuest
Fitch Solutions Group Limited	El Salvador Pharmaceuticals & Healthcare Report—Q2 2019	Fitch Solutions Group Limited	2019	08/04/2021	ProQuest
Fitch Solutions Group Limited	El Salvador Pharmaceuticals & Healthcare Report—Q3 2019	Fitch Solutions Group Limited	2019	08/04/2021	ProQuest
Fitch Solutions Group Limited	El Salvador Pharmaceuticals & Healthcare Report—Q4 2019	Fitch Solutions Group Limited	2019	08/04/2021	ProQuest
Fitch Solutions Group Limited	El Salvador Pharmaceuticals & Healthcare Report—Q4 2018	Fitch Solutions Group Limited	2018	08/04/2021	ProQuest
Egesa W, Odong R, Kalubi P, Ortiz Yamile E, Atwine D, Turyasiima M, Kiconco G, Maren M, Nduwimana M, Ssebuufu R	Preterm Neonatal Mortality and Its Determinants at a Tertiary Hospital in Western Uganda: A Prospective Cohort Study	Pediatric Health, Medicine and Therapeutics	2022	08/04/2021	ProQuest
Tickell K, Mangale D, Tornber, Belanger S, Bourdon C, Thitiri J, Timbwa M, Njirammadzi J, Voskuijl W, Chisti M, Ahmed T, Shahid A, Diallo A, Ouédrago I, Khan A, Saleem A, Arif F, Kazi Z, Mupere E, Mukisa J, Sukhtankar P, Berkley J, Walson J, Denno D	A mixed method multi-country assessment of barriers to implementing pediatric inpatient care guidelines	PLOS ONE	2019	08/04/2021	ProQuest
Nyishime M, Borg R, Ingabire W, Hedt-Gauthier B, Nahimana E, Gupta N, Hansen A, Labrecque M, Nkikabahizi F, Mutaganzwa C, Biziyaremye F, Mukayiranga C, Mwamini F, Magge H	A retrospective study of neonatal case management and outcomes in rural Rwanda post implementation of a national neonatal care package for sick and small infants	BMC Pediatrics	2018	08/04/2021	ProQuest
Puthenveettil N, Sivachalam S, Rajan S, Paul J, Kumar L	Comparison of norepinephrine and phenylephrine boluses for the treatment of hypotension during spinal anesthesia for cesarean section - A randomized controlled trial	Indian Journal of Anesthesia	2019	08/04/2021	ProQuest
Audu L, Otuneye A, Mairami A, Mukhtar-Yola M, Mshelia L	Determination of neonatal case-specific fatality rates in a tertiary health institution in North Central Nigeria	BMC Pediatrics	2021	08/04/2021	ProQuest
Lelli Chiesa P, Osman O, Aloi A, Andriani M, Benigni A, Catucci C, Giambelli P, Lisi G, Nugud F, Presutti P, Prussiani V, Racalbuto V, Rossi F, Santoponte G, Turchetta B, Salman D, Chiarelli F, Calisti A	Improving standard of pediatric surgical care in a low resource setting: The key role of academic partnership	Italian Journal of Pediatrics	2020	08/04/2021	ProQuest
Andegiorgis A, Andemariam M, Temesghen S, Ogbai L, Ogbe Z, Zeng L	Neonatal mortality and associated factors in the specialized neonatal care unit Asmara, Eritrea	BMC Public Health	2020	08/04/2021	ProQuest
Habib M, Khan K	Profile and outcomes of critically ill children in a lower middle-income country	Emergency Medicine Journal	2018	08/04/2021	ProQuest
Abu-Haydar E, Katuntu D, Bauer J, Wollen A, Eisenstein M, Sherman-Konkle J, Roche A, Ruffo M	User-centered design: Developing the reli delivery system – a low-cost, non-electric, pneumatic infusion pump	Medical Devices: Evidence and Research	2021	08/04/2021	ProQuest
Tuyisenge D, Byiringiro S, Manirakiza M, Mutsinzi R, Nshimyiryo A, Nyishime M, Hirschhorn L, Biziyaremye F, Gitera J, Beck K, Kirk C	Quality improvement strategies to improve inpatient management of small and sick newborns across All Babies Count supported hospitals in rural Rwanda	BMC Pediatrics	2021	08/04/2021	PubMed
Islam M, Zahid Rusho R, Rabiu Islam S	Design and implementation of low cost smart syringe pump for telemedicine and healthcare	First International Conference on Robotics, Electrical and Signal Processing Techniques (ICREST)	2019	08/04/2021	Web of Science
Vigneshwari N, Saranraj V, Ibrahim A, Girivasan K	Low cost, On-Demand and Intermittent Drug Delivery System through Syringe Infusion for Improving the Health of Critical Care Patients	IEEE International Conference on Intelligent Techniques in Control, Optimization and Signal Processing (INCOS)	2019	08/04/2021	IEEE Xplore
Mansour M	Design of Low Cost Smart Infusion Pump	International Conference on Computer, Control, Electrical, and Electronics Engineering (ICCCEEE)	2020	08/04/2021	IEEE Xplore
Khan M, Tariq M, Munir F, Bin Altaf M	Spur Gears and Leadscrew Based, Efficient and Flexible Infusion System Design	IEEE Biomedical Circuits and Systems Conference (BioCAS)	2018	08/04/2021	IEEE Xplore

Twenty-one of the reported healthcare facilities were primary level, 4 secondary level, and 15 tertiary level. Ninety percent of the primary, 75% of the secondary, and 50% of the tertiary healthcare facilities were located in Sub-Saharan Africa.

Three academic papers presented prototype designs from syringe pumps and for 1 an LVP. The syringe pumps were developed in Pakistan, Bangladesh, and jointly the USA and Uganda. The LVP was developed in Sudan.

The search results were published by 10 different peer reviewed Journals, 1 company, and 4 peer reviewed conference papers (Table 2).

## Discussion

### Results Interpretation

#### Global distribution of infusion pumps

The 2020 UNICEF “Every Woman, Every Child Global Strategy for Women’s, Children’s and Adolescents’ Health (2016–2030)”^3^ (2020 EWEC) global report identified that neonatal and maternal mortalities are increasingly concentrated in Sub-Saharan African countries. A similar trend was found in the results (Figure 3) from this review which showed that 6 of the 8 countries, that were identified as having none or limited access to infusion pumps, were in Sub-Saharan Africa. Additionally, 21 of the 41 healthcare facilities were located in Sub-Saharan Africa countries (Figure 2). This mirrors the concentrated effort^1,2^ to provide higher quality neonatal care to this region, as recommended in the “Survive and thrive: transforming care for every small and sick newborn” campaign.^2^

From the facilities reported in South Asia, in the search results, it was reported that infusion pumps were more widely available. Only 1 of the healthcare facilities, located in Bangladesh, reported having no access to pumps, while the other 5 had availability (Figure 3). This suggests that infusion pumps are potentially more readily available in healthcare settings in this region. This aligns with the 2020 EWEC^3^ report for neonatal mortalities, since Sub-Saharan Africa is the greatest contributor globally, however not maternal care. The report discusses how maternal mortalities are becoming increasingly concentrated in both Sub-Saharan Africa and East Asia, where 86%^3^ of maternal mortalities occurred in 2017. This may suggest that the more widespread deployment of infusion pumps, to improve quality of care, in East Asia is reducing neonatal mortalities but not having a great enough impact on maternal mortalities. However, this is a small sample size of only 6 facilities in 3 countries so cannot make this inference conclusively.

Four healthcare facilities were identified in East Asia and Pacific, all in Indonesia (Figure 3). Given all 3 were in the same country, no conclusions about the availability of infusion pumps in the whole region can be made. However, each facility was a different healthcare level and all had a sufficient number of infusion pumps. Again though, this sample size is too small to make a significant conclusion from this.

The 11 business reports all mention a tertiary-level hospital in El Salvador (Figure 3), in the Latin Americas and Caribbean region. The reports reported an insufficient number of infusion pumps at the facility. However, the availability at 1 facility cannot provide a conclusion for the whole region. The 2020 EWEC report reveals that, between 2010 and 2020, this global region had the second smallest wealth gap and the greatest healthcare coverage for women and children. This may therefore explain why only one result was identified.

No result discussed North America or Europe and Central Asia. This is unsurprising as these regions are predominantly upper-middle- and high-income countries.

#### Facility level distribution of infusion pumps

The WHO recommends the use of infusion pumps at a district hospital level (primary) and higher when treating neonates, mothers and children.^21^ The results from this search showed infusion pumps were most widely accessible in tertiary healthcare facilities, where 93% had readily or limited access to infusion pumps (Figure 3). This is unsurprising as facilities of this level should be some of the largest in each respective country, capable of providing specialist care.^22^

However, infusion pumps were only accessible in 50% of secondary level facilities, but only 4 secondary level healthcare facilities were identified. This is too few to be an accurate representation of the availability of infusion pumps at this healthcare level. Notably, 76% of primary healthcare facilities had no or limited access to infusion pumps. As previously discussed, the results are concentrated on Sub-Saharan Africa so it is unsurprising that 72% of these facilities were located in this region. However, 1 facility in Bangladesh recorded having no infusion pumps.

Although only a single result, it is only 1 of 3 primary facilities, identified in the results, located outside of Sub-Saharan Africa. This may be an indication that primary facilities across LLMICs still have variable levels of accessibility to infusion pumps.

#### Infusion pump prototypes

NEST360° and UNICEF released a Target Product Profile (TPP)^6^ detailing functional specifications for a novel syringe pump to improve infant hydration, nutrition and drug delivery. This implies that the attempts to improve the quality of therapy administered to neonates, in LLMICs, should focus on syringe pumps. This may therefore explain why 3 academic papers described the design of novel syringe pumps, while only 1 for an LVP design. The WHO also released TPPs^11^ for LVPs and syringe pumps, however they were not specific to neonates. The stringent requirements detailed in both the UNICEF/NEST360° and WHO TPPs demonstrates the importance of maintaining standards of novel syringe pumps. By preserving rigorous standards, the TPPs ensure that novel syringe pumps will improve the quality of neonatal care without compromising functionality or patient safety.

Three of the 4 designs for novel devices, from this search, were developed in LLMICs. This is in line with the work of several global bodies, including the WHO,^23,24^ which aim to encourage the local production and distribution of medical devices in LLMICs. Previous donations of medical equipment, to LLMICs, have been inappropriate for the setting, leading so disuse, storage issues and disposal costs.^23,25^ Recognizing this issue, institutions in high-income countries (HICs), such as RICE University and PATH, have begun working collaboratively to develop^26,27^ low-cost medical devices appropriate for healthcare facilities in LLMICs.

### Limitations of Results

As previously discussed, the concentration to improve neonatal care in Sub-Saharan African counties is necessary. However, this has likely limited the number of results from other regions of the world (Figure 2) creating an insufficient sample size for some regions. It is therefore difficult to draw complete conclusions about the availability of infusion pumps, for neonatal care, in these other global regions and makes comparison between them less certain.

Only 4 results discussed secondary healthcare facilities, while 21 discussed primary and 15 tertiary. It is therefore difficult to draw conclusions about the distribution of infusion pumps by healthcare facility level as too few results discussed secondary level.

The 11 quarterly business reports, published by Fitch Solutions Group Limited, were released between 2018 and 2021. Given these reports make up over 40% of the search results, they skew the reported quantity of information about the availability infusion pumps, for neonatal care, in LLMICs. However, only 1 healthcare facility is discussed in all the reports so does not have a significant impact on the overall synthesis. Yet, this does question the reliability of these reports. The same writing was used for all the reports when discussing the tertiary facility, suggesting this section of the report was not updated. Additionally, the source that the facility had a limited availability of infusion pumps was not given.

Only the results by Lelli Chiesa et al.^28^ and Tuyisenge et al.^29^ discussed the actual usability of syringe pumps. These 2 results discussed how although some syringe pumps were available, most of the facilities lacked the disposable infusion sets that are required to utilize the pumps. This means that although the pumps were available, they cannot be used due to the lack of consumables. The other results, from this search, do not disclose whether the infusion sets were also available so it cannot be conclusive that sites that have infusion pumps are utilizing them effectively. In addition to consumables, staff training on proper usage is not discussed.

Similarly, although some results state that infusion pumps are available, many do not state the extent of their availability. This may mean that despite pumps being available at a facility, there may be only a limited quantity. The results, therefore, cannot be an accurate indication of the actual usage of infusion pumps.

COVID-19 has had a global impact. The 2020 EWEC^3^ report details that almost 50% of countries surveyed reported a disruption to “essential newborn care” due to COVID-19. UNICEF,^3^ and the United Nations^30^ as a whole, recognize that the global pandemic may have affected, or even reversed, the progress made toward improving neonatal and maternal care in LLMICs. Although the effect is not yet fully quantifiable, they stipulate that the additional burden of COVID-19, in these countries, has resulted in efforts being directed away from neonatal and maternal care. This may explain why only 5 of the results were released in 2021 and 1 in 2022 (Table 2). With attention being directed away from neonatal care, less research is produced and thus the accuracy of identifying the current quality of neonatal care, in LLMICs, is reduced.

### Limitations of Method

The method is limited by the search terms themselves. By including the terms “*neonatal*,” “*newborn*,” “*pediatric*,” and “*pediatric*,” search results are better filtered so that results discussing the subject are more easily identifiable. However, this has potentially created a sample frame error as it does not necessarily give a fair representation of the actual availability of infusion pumps for neonates in LLMICs globally. By using this search strategy, the search presented results of healthcare facilities where a concerted effort to deploy infusion pumps, to treat neonates, has been made. As a result, this does not consider healthcare facilities where there has been no effort to establish specialist neonatal care yet often provide neonatal care. This means that the actual availability of infusion pumps per birth, in each region, cannot be identified by this search.

Furthermore, this search did not consider the provenance of the search results. Since the selected sample size was only 28, the quality of the results was not considered extensively. The lack of included search results meant that all the results were needed to perform the synthesis. Similarly, the type of results were not considered in the exclusion criteria. Opinion pieces, narrative reviews and other non-original research would have been included using this method. This may mean that the information within such papers may be outside of the date exclusion criteria although the paper itself meets the criteria. However, this limitation did not affect the results as all the search results were academic papers of original research or quarterly business reports.

Since only 1 author performed the search, a population error may have occurred. Only 4 databases were searched, meaning that only 9130 article titles were rapidly screened and only 230 read in full.

There is a potential bias in the exclusion criteria. There may be bias when considering what the reviewer considered adequate English, theoretically creating a sampling error. To reduce this possible error, the reviewer used the English language filter on each database and included results where the sentences were comprehensible, and the information could be understood. However, no result was excluded due to inadequate translation into English.

### Implications

Now the issue of the lack of quality, specialist neonatal care has been identified, recommended changes^1-3^ must be implemented. This should include the wider use of infusion pumps to aid in providing precise drug and fluid delivery to neonates. The deployment of the “All Babies Count” (ABC) improvement strategy by Tuyisenge et al.^29^ in Rwanda, including the increased use of infusion pumps, led to a significant reduction in neonatal mortality during the 18 months trial period. Similar improvement packages should be deployed in more primary and secondary level healthcare facilities across Sub-Saharan African countries to close the “survival gap” and help meet the third SDG.

Although this review may give an indication on the global availability of infusion pumps in LLMICs, it cannot give an indication on the coverage of quality neonatal care. The results from this search have shown the distribution and accessibility of infusion pumps in the identified healthcare facilities. However, they have not detailed what portions of each country’s population is covered by the improved quality of neonatal care. More research into the global coverage of specialist, neonatal services should be performed to ensure that neonatal mortalities are reduced unilaterally.

As previously discussed, UNICEF and the United Nations are concerned^3,30^ about the impact of COVID-19 on the continual improvement to neonatal, maternal and pediatric care in LLMICs. Assessments of recently implemented neonatal care improvement strategies should be performed quickly so that the extent of the potential regression, of these services, can be evaluated and corrected if necessary. This is particularly true for medical equipment like infusion pumps. Infusion pumps can also be used to treat adult patients so may have been repurposed for adult care during the COVID-19 pandemic. Only a revaluation of previously existing services will reveal the extent that this has occurred.

## Conclusion

Efforts to identify and reduce the “survival gap”^2^ have been concentrated on LLMICs in Sub-Saharan Africa; the region with the highest rate of neonatal and maternal mortalities. The results from this search showed that infusion pumps had limited deployment in this global region compared to the other 3 discussed. This suggests that there must be an increased effort to deploy suitable neonatal care improvement packages across the Sub-Saharan Africa region, including improved accessibility to infusion pumps.

Access to infusion pumps could be improved by deploying pumps designed specifically to overcome the challenges of providing quality healthcare in LLMICs^18^; such as cost and unreliable utilities. Other types of medical devices, designed for use in LLMICs, have been proven effective in helping to achieve this.^30-33^ Several designs of infusion pumps have already been developed,^34-38^ however none have been widely deployed yet. Trialing the use of specifically designed infusion pumps could help improve the accessibility of quality of infant hydration, nutrition and drug delivery.

This search returned limited results that discussed regions outside of Sub-Saharan Africa. This, combined with the effect that COVID-19 had on healthcare systems globally, calls into question the coverage of quality neonatal care, including access to infusion pumps, in LLMICs in other global regions. Rapid assessments of newly established neonatal services, in all regions of the world, should be conducted to measure their robustness and continuing effectiveness to help reduce neonatal mortalities globally.

These proposals aim to continue the campaign to improve neonatal care globally and reduce the rate of mortality of sick and premature newborns.
